# Preoperative sustained C-reactive protein elevation shortens patient survival after liver resection for perihilar cholangiocarcinoma: A retrospective cohort study with propensity score analyses

**DOI:** 10.1097/MD.0000000000051245

**Published:** 2026-10-30

**Authors:** Yuki Fujii, Tatsuhiko Kakisaka, Hirofumi Kamachi, Hiroki Nakamoto, Tatsuya Orimo, Akihisa Nagatsu, Takeshi Aiyama, Kazuki Wakizaka, Shunsuke Shichi, Ken Imaizumi, Akinobu Taketomi

**Affiliations:** a Department of Gastroenterological Surgery 1, Hokkaido University Graduate School of Medicine, Sapporo, Hokkaido, Japan; b Department of Surgery, Fukuoka Wajiro Hospital, Fukuoka, Japan; c Department of Biostatistics, Hokkaido University Graduate School of Medicine, Sapporo, Hokkaido, Japan.

**Keywords:** C-reactive protein, inflammatory markers, Klatskin tumor, overall survival, perihilar cholangiocarcinoma

## Abstract

Preoperative inflammation occurs frequently in perihilar cholangiocarcinoma (P-CCA). However, the prognostic significance of maximum preoperative inflammatory markers and the necessity of monitoring preoperative inflammation in P-CCA remain underexplored. We aimed to clarify the clinical impact of preoperative inflammation on survival in P-CCA patients by assessing changes in C-reactive protein (CRP) levels (maximum and 1–2 days before surgery), thereby informing optimal management strategies for P-CCA. This retrospective study included patients with P-CCA who underwent extrahepatic bile duct resection and hepatectomy at the Department of Gastroenterological Surgery 1, Hokkaido University Hospital, between 2000 and 2019. In addition to inflammatory markers measured 1 to 2 days before surgery (referred to as “values prior to surgery”), maximum preoperative CRP was also recorded. The primary outcome was overall survival (OS), and a multivariate Cox regression model was used to assess clinicopathological factors associated with OS. One-to-one propensity score matching was performed to match tumor characteristics and maximum preoperative CRP levels between the alleviated and sustained CRP groups. We identified 100 patients, of whom 89 (89%) had a clinically meaningful preoperative inflammatory response; 39% had cholangitis, and 65% underwent percutaneous transhepatic portal embolization. The median OS was 34.5 months (interquartile range [IQR]: 16.6–65.2), and the 5-year OS rate was 34.4%. In the univariate and multivariate analyses, elevated CRP prior to surgery was associated with shorter OS (multivariate hazard ratio [HR], 1.22; 95% confidence interval [CI], 1.04–1.42), whereas no association was found between the maximum preoperative CRP and OS. Additionally, preoperative cholangitis (HR, 1.91; 95% CI, 1.17–3.14) and an elevated neutrophil-to-lymphocyte ratio (HR, 1.10; 95% CI, 1.00–1.17) were associated with a worse outcome in the univariate analyses. Furthermore, after propensity score matching, patients whose CRP normalized before surgery (alleviated CRP group) had better OS than those with sustained CRP elevation (sustained CRP group; median OS: 49.8 vs 22.6 months, respectively; *P* = .017). This study revealed the negative prognostic impact of preoperative inflammation in P-CCA. Notably, persistent elevation of CRP prior to surgery predicted poor prognosis in P-CCA, suggesting that waiting for CRP normalization may improve survival outcomes.

## 1. Introduction

Perihilar cholangiocarcinoma (P-CCA), or Klatskin tumor, is one of the most devastating cancers, with a reported 5-year overall survival (OS) rate of 10% and a median survival of 7 months.^[1]^ Surgical resection is considered the only curative treatment, increasing the 5-year OS rate to 40% and the median survival to 35 months.^[2-4]^ However, surgical removal is challenging, with only one-third of patients being eligible for successful resection.^[1,5]^ The difficulty lies in the extensive nature of the surgery, which often requires major hepatectomies, and the complexity of preoperative management, including diagnostic difficulties and the necessity for a multimodal approach involving endoscopy. Furthermore, concomitant inflammation – frequently due to cholangitis – complicates treatment, and cholangitis affects approximately 20% of patients.^[2,4,6]^ In addition to cholangitis, obstructive jaundice, endoscopic retrograde cholangiopancreatography, and percutaneous transhepatic portal embolization (PTPE) can also cause preoperative inflammation, further exacerbating the complexity of P-CCA management.

Numerous studies have linked elevated inflammatory markers to poorer outcomes in various cancers, including pancreatic, esophageal, and ovarian cancers.^[7-10]^ This association also holds for intrahepatic cholangiocarcinomas.^[11,12]^ Notably, both cancer-associated and noncancerous inflammation negatively affect cancer patients’ survival.^[13,14]^ Similarly, preoperative inflammation is an important predictor of poor short- and long-term outcomes in P-CCA. In several studies, preoperative cholangitis increased morbidity after hepatectomies for P-CCA and impaired OS or disease-free survival.^[6,15-17]^ Moreover, elevated inflammatory markers, including the Glasgow prognostic score, neutrophil-to-lymphocyte ratio (NLR), platelet-to-lymphocyte ratio (PLR), and C-reactive protein (CRP), are significantly associated with worse patient survival.^[2,4,18]^

Despite multiple factors contributing to inflammation in P-CCA, there is no consensus on the optimal management to control preoperative inflammation. Surgical timing remains unstandardized in many institutions, with decisions often based on jaundice severity or serum total bilirubin (T-bili) levels, despite the lack of justification for routine preoperative biliary drainage.^[19,20]^ Given that preoperative inflammation may be modifiable within a short period, the extent to which inflammation should be controlled remains debated. Furthermore, while elevated inflammatory markers are associated with poor prognosis in resectable P-CCA, they are typically measured only days before surgery. Therefore, the impact of maximum preoperative inflammation on patient survival is unclear. From this perspective, we hypothesized that maximum preoperative inflammatory markers could be crucial prognostic factors for patients with resectable P-CCA, and sufficient alleviation of systemic inflammation before surgery may improve long-term outcomes compared to sustained inflammation. To investigate this hypothesis, we analyzed laboratory data and histopathological tumor characteristics from our institute, assessing their associations with the survival of P-CCA patients.

## 2. Materials and methods

We conducted a single-institution retrospective cohort study by analyzing electronic medical records from the Department of Gastroenterological Surgery 1 at Hokkaido University Hospital. Patients who underwent extrahepatic bile duct resection with hepatectomy between 2000 and 2019 were included. Those with gallbladder cancer, intrahepatic cholangiocarcinoma, or extrahepatic bile duct resection without hepatectomy were excluded from the study. This study was approved by the Institutional Review Board of Hokkaido University Hospital (#022-0221) and conducted in accordance with the Declaration of Helsinki and the Uniform Requirements for Manuscripts Submitted to Biomedical Journals. Because of the retrospective nature of the study and the use of anonymized existing clinical records, the requirement for written informed consent was waived by the Institutional Review Board, and an opt-out method was used to provide patients with the opportunity to decline participation.

At our department, the resectability of P-CCA was determined by patient status (performance status score ≤ 2) and tumor progression evaluated by preoperative imaging using endoscopic retrograde cholangiopancreatography, computed tomography, and magnetic resonance imaging/cholangiopancreatography, combined with intraoperative findings. Briefly, tumors without major vascular invasion (superior mesenteric or common hepatic artery) or distant metastases were considered resectable. Surgical timing was determined by T-bili levels, targeting < 2.0 mg/dL (34.2 µmol/L) on the day of surgery, with no signs of progressive cholangitis or sepsis, defined as negative blood cultures and the absence of systemic inflammatory response syndrome per Sepsis-3 guidelines.^[21]^

### 2.1. Variables

The data were analyzed to identify factors associated with preoperative inflammatory markers and survival outcomes. The primary endpoint was OS, and we used a multivariate Cox regression model to assess relationships between clinicopathological characteristics and OS.

Preoperative T-bili, tumor markers, and inflammatory markers – namely, white blood cells, neutrophils, lymphocytes, platelets, albumin (Alb), and CRP – were collected 1 to 2 days before surgery. Where more than 1 measurement was available within this window, the value closest to surgery was used, and no averaging or imputation was performed (“values prior to surgery”). Maximum preoperative CRP, white blood cell and T-bili were defined as the single highest values recorded at any time between the date of diagnosis and the day of surgery, identified by screening all measurements available in the electronic medical record over that interval. Biliary obstruction, drainage procedures, cholangitis, and PTPE each cause a sharp rise in CRP that typically peaks within days and falls over 1 to 3 weeks once treated; the maximum preoperative CRP therefore reflects the largest such event, whereas the CRP prior to surgery represents residual inflammation at the time of surgery. Because testing frequency followed clinical need rather than a fixed protocol, the number of measurements per patient varied.

Other baseline variables comprised age, sex, body mass index, American Society of Anesthesiologists classification, incidence of preoperative cholangitis, PTPE, surgical details (type of hepatectomy, pancreaticoduodenectomy [PD] and/or vascular resection, remnant liver volume, estimated blood loss, and operative time), pathological stage (Union for International Cancer Control tumor-node-metastasis [TNM] staging system, 8th edition), pathological findings (pathological differentiation, lymph vessel invasion, microvascular invasion, perineural invasion, and residual tumor [R status]), postoperative complications categorized according to the Clavien–Dindo classification system, and the durations of OS and recurrence-free survival (RFS). Patients with residual tumors localized solely to the mucosa (carcinoma in situ) were considered to have R0 residual tumors.^[22]^

### 2.2. Statistical analysis

Patient characteristics were summarized overall and stratified by sustained and alleviated CRP groups using descriptive statistics. Patients whose maximum preoperative CRP never reached 1.0 mg/dL (10 mg/L) – the threshold used to define systemic inflammation in inflammation-based prognostic scores^[4]^ – were considered not to have mounted a clinically meaningful inflammatory response and were excluded from the group comparison, both before and after propensity score matching (PSM). Among the remaining patients, the alleviated CRP group was defined by a CRP prior to surgery below the institutional upper limit of normal (<0.5 mg/dL [5 mg/L]) and the sustained CRP group by a CRP prior to surgery at or above that limit (≥0.5 mg/dL [5 mg/L]). The cutoff value of CRP was defined by the institutional standard.^[18,23]^

Continuous variables were expressed as mean ± standard deviation or as median with interquartile range (IQR). Differences between categorical variables were compared using the chi-square or Fisher’s exact test, and differences between continuous variables were compared using the Student *t* test. Univariate and multivariate Cox regression models were applied to assess relationships between clinicopathological characteristics and OS. In these models, *p*-values for all variables were determined by the Wald test, and hazard ratios for continuous variables were reported per 1-unit change. Univariate and multivariate logistic regression models evaluated associations between recurrence and patient/tumor characteristics. OS was defined as the duration from diagnosis to last contact or death from any cause, and RFS as the duration from surgery to the date of recurrence confirmed by imaging studies or biopsy. Survival curves and 5-year OS values were generated using the Kaplan–Meier method, with the log-rank test used to compare the survival curves between the 2 patient groups. All analyses were performed on complete cases; for each model, patients with a missing value for any variable in that model were excluded, and no imputation was performed. Missing values did not exceed 10% for any variable, and no assumption was made that they were missing at random. The significance level for all statistical tests was set at *P* < .05 (2-tailed test). All statistical analyses were performed using JMP Pro 16.2.0 software (SAS Institute Inc., Cary).

PSM was performed at a 1:1 ratio to balance differences between the sustained and alleviated CRP groups. PSM variables included age, sex, maximum preoperative CRP, Bismuth classification, regional lymph node metastases, pathological T and TNM classification, tumor differentiation, and the presence of perineural, lymph vessel, or microvascular invasion. The caliper was set to 0.20. Covariate balance between groups was assessed using standardized mean differences. SMDs were calculated using pooled standard deviations for continuous and binary variables and a multinomial extension for variables with more than 2 categories.

Several sensitivity analyses were performed. First, CRP prior to surgery and maximum preoperative CRP were modeled per standard deviation, per doubling, on the natural logarithmic scale, and in tertiles. Second, the effect of the CRP group was examined in the matched cohort using a multivariate Cox regression model that included PD performed at surgery and R status. Third, to address confounding by the cause of inflammation, the analyses were repeated with adjustment for and stratification on preoperative cholangitis.

## 3. Results

### 3.1. Patient demographic and tumor characteristics

We identified 100 patients who met the study criteria (Table 1). The mean age was 69.9 ± 7.4 years, and 73% were male. The mean body mass index was 22.3 ± 3.0 kg/m^2^, and 7 patients were classified as American Society of Anesthesiologists classification grade 3 at surgery. The majority of the patients (89%) had a clinically meaningful inflammatory response, defined as a maximum preoperative CRP of at least 1.0 mg/dL (10 mg/L). Preoperative endoscopic and/or percutaneous transhepatic biliary drainage was performed in 92% of the patients, while 39% had cholangitis, and 65% underwent PTPE. The mean maximum preoperative white blood cell and CRP values were 10,158.0 ± 4911.2/µL and 7.89 ± 6.03 mg/dL, decreasing to 5446.0 ± 1787.2/µL and 1.19 ± 1.85 mg/dL, respectively, prior to surgery. The mean decline in CRP from the peak to the value prior to surgery was 6.71 ± 5.43 mg/dL, corresponding to a mean reduction of 78.3 ± 29.1% of the peak, and the maximum and prior-to-surgery values were only moderately correlated (Spearman ρ = 0.47; *P* < .001). T-bili also declined from a peak of 3.35 ± 3.54 mg/dL to 1.01 ± 0.52 mg/dL at surgery. The median interval from diagnosis to surgery was 65.0 days (IQR: 50.3–83.0).

**Table 1:** Patient demographics and tumor characteristics.

Variable	Values All (n = 100)
Age	69.9 ± 7.4
Sex (female, %)	27 (27%)
BMI (kg/m^2^)	22.3 ± 3.0
ASA classification (1/2/3)	8/85/7
Preoperative drainage (none/PTBD/EBD/both/UNK)	7/21/61/10/1
Preoperative cholangitis (Y/N/UNK)	39/51/10
PTPE (Y/N/UNK)	65/34/1
WBC (preoperative maximum)	10,158.0 ± 4911.2
WBC (prior to surgery)	5446.0 ± 1787.2
Neutrophils	3209.3 ± 1556.9
Lymphocytes	1322.1 ± 584.1
Platelets	234408 ± 94306.3
CRP (preoperative maximum)	7.89 ± 6.03
CRP (prior to surgery)	1.19 ± 1.85
Alb	3.64 ± 0.4
T-bili (preoperative maximum)	3.35 ± 3.54
T-bili (prior to surgery)	1.01 ± 0.52
DUPAN-2	480.1 ± 798.9
CEA	4.00 ± 3.59
CA 19-9	209.4 ± 582.8
Span-1	208.6 ± 1142.2
Days from diagnosis to surgery	65.0 (50.3–83.0)
Type of hepatectomy (right/left/other)	65/29/6
PD (Y/N)	12/88
Remnant liver volume (mL)	604.4 ± 166.8
Vascular resection/reconstruction (Y/N)	30/70
Estimated blood loss (mL)	1546.4 ± 1549.0
Operative time (minutes)	644.5 ± 147.9
Bismuth classification (I/II/III/IV)	1/13/59/27
Pathological T stage (1/2/3/4)	2/60/14/24
Pathological differentiation (well/mod/poor/other)	9/39/49/3
Lymph vessel invasion (Y/N/UNK)	47/47/6
Microvascular invasion (Y/N/UNK)	61/33/6
Perineural invasion (Y/N/UNK)	91/3/6
R status (R0/R1/R2)	60/39/1
Pathological N stage (0/1)	46/54
Pathological TNM stage (1/2/3/4)	2/34/44/20
Postoperative complication (none/Clavien–Dindo classification I/II/III/IV/V)	25/3/13/53/1/5
Length of postoperative hospital stay (d)	36.5 (27.3–62.8)
Adjuvant chemotherapy (Y/N/UNK)	59/37/4
Recurrence (Y/N)	68/32
Overall survival (mo)	34.5 (16.6–65.2)
Recurrence-free survival (mo)	19.6 (8.8–52.4)

Values are presented as mean ± standard deviation or median (IQR).

Alb = albumin, ASA = American Society of Anesthesiologists, BMI = body mass index, CA 19-9 = carbohydrate antigen 19-9, CEA = carcinoembryonic antigen, CRP = C-reactive protein, DUPAN-2 = Duke pancreatic monoclonal antigen type 2, EBD = endoscopic biliary drainage, IQR = interquartile range, N = no, N stage = lymph node stage, PD = pancreatoduodenectomy, PTBD = percutaneous transhepatic biliary drainage, PTPE = percutaneous transhepatic portal embolization, R status = residual tumor status, Span-1 = s-pancreas antigen-1, T stage = tumor stage, T-bili = total bilirubin, TNM = tumor-node-metastasis, UNK = unknown, WBC = white blood cell, Y = yes.

Right hepatectomy accompanied by extrahepatic bile duct resection was performed in 65% of the patients, while left hepatectomy was performed in 29%. The mean remnant liver volume was 604.4 ± 166.8 mL. PD was performed simultaneously with hepatectomy in 12% of the patients, and vascular resection and reconstruction were performed in 30%. Most tumors were Bismuth III (59%) and pathological stage T2 (60%). Lymph vessel, microvascular, or perineural invasion was observed in 47%, 61%, or 91% of the patients, respectively. R0 resection was achieved in 60% of the patients, of whom 10 (10%) had residual carcinoma in situ, while 40% had residual tumors, including 1 patient (1%) with R2 residual tumor.

Postoperative complications occurred in 75% of the patients, and the postoperative mortality rate was 5%. Fifty-three patients experienced Clavien–Dindo grade III complications, primarily pleural fluid collection requiring aspiration or drainage. The median postoperative hospital stay was 36.5 days (IQR: 27.3–62.8). Adjuvant chemotherapy was administered to 59% of the patients, in accordance with standard guideline regimens. Recurrence was observed in 68% of the patients, and the median OS and RFS reached 34.5 months (IQR: 16.6–65.2) and 19.6 months (IQR: 8.8–52.4), respectively.

### 3.2. Survival analyses

A total of 100 patients were included in the uni- and multivariate Cox regression analyses. During this period, 77 patients (77%) died, and the median follow-up time for surviving patients was 61 months (IQR: 32–74). The median OS was 34.5 months (IQR: 16.6–65.2), and the 5-year OS rate was 34.4%.

The results of the univariate and multivariate Cox regression models for OS are shown in Table 2. In the univariate analyses, patients with preoperative cholangitis experienced significantly shorter OS compared with those without preoperative cholangitis (HR, 1.91; 95% confidence interval [CI], 1.17–3.14). Similarly, elevated maximum preoperative T-bili was also associated with shorter OS (HR, 1.07; 95% CI, 1.01–1.12). In contrast, PTPE did not shorten OS. Among the inflammatory indicators, elevated NLR (HR, 1.10; 95% CI, 1.00–1.17), CRP prior to surgery (HR, 1.22; 95% CI, 1.06–1.38), and CRP/Alb ratio (HR, 1.79; 95% CI, 1.07–2.77) were all associated with significantly shorter OS, with every 1-unit increase in these values. However, maximum preoperative CRP levels were not associated with shorter OS.

**Table 2:** Univariate and multivariate cox regression analyses of overall survival (n = 100).

Variable		Univariate				Multivariate			
		Hazard ratio	95% CI		*P*-value	Hazard ratio	95% CI		*P*-value
Age		1.02	0.99	1.06	.148	1.07	1.03	1.11	<.001
Sex	Male	Reference			.375	Reference			.009
	Female	0.79	0.46	1.33		0.49	0.27	0.90	
BMI		0.95	0.88	1.03	.228				
ASA	1	Reference			.361				
	2/3	1.48	0.63	3.46					
Preoperative cholangitis	No	Reference			.010				
	Yes	1.91	1.17	3.14					
PTPE	No	Reference			.987				
	Yes	1.03	0.64	1.65					
Preoperative drainage	None	Reference			.067				
	Percutaneous	0.87	0.31	2.42					
	Endoscopic	1.32	0.52	3.34					
	Both	2.75	0.94	8.09					
WBC (preoperative maximum)		1.00	1.00	1.00	.292				
WBC (prior to surgery)		1.00	1.00	1.00	.625				
NLR		1.10	1.00	1.17	.018				
PLR		1.00	1.00	1.00	.069				
CRP (preoperative maximum)		1.02	0.99	1.07	.203				
CRP (prior to surgery)		1.22	1.06	1.38	.002	1.22	1.04	1.42	.022
CRP/Alb		1.79	1.07	2.77	.015				
T-bili (preoperative maximum)		1.07	1.01	1.12	.018				
T-bili (prior to surgery)		1.65	1.07	2.42	.016				
DUPAN-2		1.00	1.00	1.00	.421				
CEA		1.02	0.96	1.07	.576				
CA 19-9		1.00	1.00	1.00	.740				
Span-1		1.00	1.00	1.00	.567				
Type of hepatectomy	Right	Reference			.834				
	Left	1.00	0.61	1.65					
	Other	1.32	0.53	3.33					
PD	No	Reference			.173				
	Yes	1.63	0.81	3.29					
Remnant liver volume		1.00	1.00	1.00	.669				
Vascular resection	No	Reference			.008	Reference			.059
	Yes	1.95	1.19	3.19		1.76	0.98	3.16	
Estimated blood loss (L)		1.31	1.14	1.46	<.001				
Operative time (hours)		1.16	1.06	1.27	<.001				
Bismuth classification	I–III	Reference			.009	Reference			.610
	IV	1.91	1.17	3.09		1.15	0.67	1.99	
Pathological T stage	1/2	Reference			<.001				
	3/4	2.38	1.47	3.84					
Pathological differentiation	Well/mod	Reference			.624				
	Poor	0.98	0.62	1.55					
	Other	0.45	0.11	2.14					
Lymph vessel invasion	No	Reference			.187				
	Yes	1.38	0.86	2.22					
Microvascular invasion	No	Reference			.091				
	Yes	1.55	0.93	2.56					
Perineural invasion	No	Reference			.928				
	Yes	0.94	0.23	3.84					
R status	R0	Reference			.097	Reference			.030
	R1/R2	1.47	0.93	2.32		1.74	1.06	2.88	
Pathological N stage	0	Reference			.012				
	1	1.81	1.14	2.86					
Pathological TNM stage	1/2	Reference			<.001	Reference			.001
	3/4	2.56	1.54	4.28		2.55	1.45	4.48	
Postoperative complications	<CD III	Reference			.214				
	≥CD III	1.37	0.83	2.26					
Adjuvant chemotherapy	No	Reference			.018	Reference			<.001
	Yes	0.56	0.35	0.90		0.34	0.20	0.58	

Alb = albumin, ASA = American Society of Anesthesiologists, BMI = body mass index, CA 19-9 = carbohydrate antigen 19-9, CD = Clavien–Dindo, CEA = carcinoembryonic antigen, CI = confidence interval, CRP = C-reactive protein, DUPAN-2 = Duke pancreatic monoclonal antigen type 2, mod = moderately differentiated, NLR = neutrophil-to-lymphocyte ratio, PD = pancreatoduodenectomy, PLR = platelet-to-lymphocyte ratio, poor = poorly differentiated, PTPE = percutaneous transhepatic portal embolization, R status = residual tumor status, Span-1 = s-pancreas antigen-1, T stage = tumor stage, T-bili = total bilirubin, TNM = tumor-node-metastasis, WBC = white blood cell, well = well differentiated.

In the multivariate analyses, we included age, sex, CRP, vascular resection during surgery, Bismuth classification, R status, pathological TNM stage, and adjuvant chemotherapy. These factors were selected based on previous research suggesting their relevance to patient prognosis.^[2,17,18,24-26]^ The results showed that high CRP (HR, 1.22; 95% CI, 1.04–1.42), older age (HR, 1.07; 95% CI, 1.03–1.11), advanced pathological TNM stage (HR, 2.55; 95% CI, 1.45–4.48), and residual tumor (R1 or R2 status; HR, 1.74; 95% CI, 1.06–2.88) were associated with shorter OS, whereas female sex (HR, 0.49; 95% CI, 0.27–0.90) and adjuvant chemotherapy (HR, 0.34; 95% CI, 0.20–0.58) were associated with longer OS. We also performed sensitivity analyses using alternative parameterizations of both the maximum preoperative CRP and the CRP prior to surgery; the findings were essentially unchanged across all specifications, with elevated CRP prior to surgery remaining associated with shorter OS and the maximum preoperative CRP remaining unassociated with it (Tables S1 and S2, Supplemental Digital Content 1).

### 3.3. Clinicopathological factors associated with recurrence

A total of 100 patients were included in the univariate and multivariate logistic regression analyses. Among these patients, 21% (n = 21) experienced locoregional recurrence, while 47% (n = 47) had distant metastases – namely, to the peritoneum (15%), liver (12%), extra-regional lymph nodes (6%), and other locations (14%; Table S3, Supplemental Digital Content 2).

The results of the univariate and multivariate logistic regression analyses for recurrence are summarized in Table 3. In the univariate analyses, high maximum preoperative T-bili levels, elevated preoperative T-bili prior to surgery, vascular resection during surgery, and advanced pathological T and TNM stages were associated with a higher incidence of recurrence. The multivariate analysis revealed that high T-bili prior to surgery (odds ratio, 9.86; 95% CI, 2.28–62.61) and vascular resection during surgery (odds ratio, 3.64; 95% CI, 1.15–14.09) were independently associated with an increased risk of recurrence.

**Table 3:** Univariate and multivariate logistic regression analyses of recurrence (n = 100).

Variable		Univariate				Multivariate			
		Odds ratio	95% CI		*P*-value	Odds ratio	95% CI		*P*-value
Age (yr)		1.01	0.96	1.07	.177				
Sex	Male	Reference			.207				
	Female	1.94	0.73	5.81					
BMI		0.65	0.05	7.84	.728				
ASA	1	Reference			.729				
	2/3	1.30	0.25	5.68					
Preoperative cholangitis	No	Reference			.245				
	Yes	1.72	0.7	4.42					
PTPE	No	Reference			.180				
	Yes	0.53	0.20	1.31					
Preoperative drainage	None	Reference			.792				
	Percutaneous	0.65	0.08	3.87					
	Endoscopic	0.82	0.11	4.18					
	Both	1.6	0.15	17.27					
WBC (preoperative maximum)		1.00	1.00	1.00	.316				
WBC (prior to surgery)		1.00	1.00	1.00	.629				
NLR		1.20	0.96	1.68	.214				
PLR		1.00	0.99	1.01	.269				
CRP (preoperative maximum)		1.01	0.95	1.10	.711				
CRP (prior to surgery)		1.12	0.88	1.51	.412				
CRP/Alb		1.16	0.52	3.01	.722				
T-bili (preoperative maximum)		1.34	1.08	1.78	.023				
T-bili (prior to surgery)		10.03	2.59	56.99	<.001	9.86	2.28	62.61	<.001
DUPAN-2		1.00	1.00	1.00	.255				
CEA		1.00	0.88	1.13	.897				
CA 19-9		1.00	1.00	1.00	.250				
Span-1		1.00	1.00	1.00	.620				
Type of hepatectomy	Right	Reference			.635				
	Left	0.98	0.39	2.62					
	Other	0.44	0.08	2.58					
PD	No	Reference			.916				
	Yes	1.07	0.27	3.71					
Remnant liver volume		1.00	1.00	1.00	.483				
Vascular resection	No	Reference			.013	Reference			.027
	Yes	4.33	1.49	15.88		3.64	1.15	14.09	
Estimated blood loss (L)		0.98	0.75	1.34	.894				
Operative time (hours)		1.19	0.99	1.45	.064				
Bismuth classification	I–III	Reference			.546				
	IV	1.34	0.53	3.62					
Pathological T stage	1/2	Reference			.045				
	3/4	2.51	1.02	6.70		2.05	0.79	5.34	.138
Pathological differentiation	Well/mod	Reference			.313				
	Poor	1.52	0.64	3.67					
	Other	0.27	0.01	3.06					
Lymph vessel invasion	No	Reference			.274				
	Yes	1.62	0.68	3.93					
Microvascular invasion	No	Reference			.332				
	Yes	1.55	0.63	3.79					
Perineural invasion	No	Reference			.989				
	Yes	1.02	0.04	11.02					
R status	R0	Reference			.726				
	R1/R2	1.17	0.50	2.82					
Pathological N stage	0	Reference			.328				
	1	1.52	0.66	3.58					
Pathological TNM stage	1/2	Reference			.016				
	3/4	2.92	1.23	7.11					
Postoperative complications	<CD III	Reference			.930				
	≥CD III	1.04	0.44	2.44					
Adjuvant chemotherapy	No	Reference			.516				
	Yes	0.75	0.31	1.82					

Alb = albumin, ASA = American Society of Anesthesiologists, BMI = body mass index, CA 19-9 = carbohydrate antigen 19-9, CD = Clavien–Dindo, CEA = carcinoembryonic antigen, CI = confidence interval, CRP = C-reactive protein, DUPAN-2 = Duke pancreatic monoclonal antigen type 2, mod = moderately differentiated, N stage = lymph node stage, NLR = neutrophil-to-lymphocyte ratio, PD = pancreatoduodenectomy, PLR = platelet-to-lymphocyte ratio, poor = poorly differentiated, PTPE = percutaneous transhepatic portal embolization, R status = residual tumor status, Span-1 = s-pancreas antigen-1, T stage = tumor stage, T-bili = total bilirubin, TNM = tumor-node-metastasis, WBC = white blood cell, well = well differentiated.

### 3.4. PSM analyses for the alleviated or sustained CRP groups

To investigate the extent to which preoperative inflammation should be controlled in P-CCA patients, we divided the patients into the alleviated CRP group and the sustained CRP group according to the definition described in the Methods section. Eleven patients whose maximum preoperative CRP never reached 1.0 mg/dL were excluded from the group comparison, leaving 89 patients. We used PSM to minimize the influence of tumor characteristics and adjust for the degree of systemic inflammation, as described in the Methods section. Although sex showed the largest residual imbalance among the matched covariates (absolute SMD, 0.24; women, 5/30 vs 8/30), other clinically important covariates associated with survival were well balanced after PSM (Table 4).

**Table 4:** Patient demographic and tumor characteristics in the alleviated or sustained CRP groups before and after propensity score matching.

Variable	Before matching				After matching			
	Alleviated CRP group (n = 44)	Sustained CRP group (n = 45)	*P*-value	SMD	Alleviated CRP group (n = 30)	Sustained CRP group (n = 30)	*P*-value	SMD
Age	70.8 ± 6.3	69.4 ± 7.6	.358	0.20	70.2 ± 6.0	70.1 ± 7.1	.969	0.01
Sex (female, %)	8 (18.2%)	13 (28.9%)	.232	−0.25	5 (16.7%)	8 (26.7%)	.532	−0.24
BMI	22.5 ± 3.0	22.1 ± 3.2	.516	0.14	22.8 ± 3.4	22.1 ± 3.4	.458	0.20
ASA (1/2/3)	1/39/4	4/39/2	.391	0.29	0/27/3	2/26/2	.671	0.38
Preoperative drainage (none/PTBD/EBD/both/UNK)	0/11/26/6/1	3/8/30/4/0	.299	0.45	0/8/18/4/0	3/4/21/2/0	.163	0.62
Preoperative cholangitis (Y/N/UNK)	17/23/4	20/19/6	.434	−0.18	11/16/3	14/14/2	.490	−0.19
PTPE (Y/N/UNK)	33/10/1	32/13/0	.547	0.13	26/4/0	21/9/0	.209	0.41
WBC (preoperative maximum)	10045.5 ± 3647.1	11,206.7 ± 5979.9	.273	−0.23	10,100.0 ± 3498.2	10,933.3 ± 6943.9	.560	−0.15
WBC (prior to surgery)	5034.1 ± 1434.1	5728.9 ± 2086.5	.071	−0.39	5033.3 ± 1523.2	5526.7 ± 2067.8	.297	−0.27
NLR	2.27 ± 1.07	4.18 ± 3.63	.006	−0.71	2.30 ± 1.23	4.75 ± 4.20	.010	−0.79
PLR	188.0 ± 118.0	252.4 ± 143.4	.047	−0.49	186.7 ± 127.9	271.3 ± 150.8	.041	−0.60
CRP (preoperative maximum)	7.14 ± 5.40	10.47 ± 5.64	.006	−0.60	7.94 ± 5.92	8.52 ± 4.25	.664	−0.11
CRP (prior to surgery)	0.17 ± 0.13	2.40 ± 2.21	<.001	−1.42	0.17 ± 0.13	1.93 ± 1.71	<.001	−1.44
Alb	3.79 ± 0.36	3.44 ± 0.32	<.001	1.05	3.81 ± 0.39	3.46 ± 0.28	<.001	1.06
CRP/Alb	0.043 ± 0.035	0.672 ± 0.654	<.001	−1.36	0.042 ± 0.036	0.565 ± 0.533	<.001	−1.38
T-bili (preoperative maximum)	3.62 ± 4.04	3.63 ± 3.28	.996	−0.00	4.05 ± 4.60	3.51 ± 3.43	.610	0.13
T-bili (prior to surgery)	0.89 ± 0.32	1.17 ± 0.66	.012	−0.55	0.90 ± 0.34	1.22 ± 0.61	.015	−0.64
DUPAN-2	450.5 ± 822.6	519.8 ± 821.4	.699	−0.08	602.8 ± 960.0	586.9 ± 977.3	.951	0.02
CEA	3.21 ± 1.70	4.96 ± 4.87	.027	−0.48	3.44 ± 1.60	4.68 ± 4.48	.159	−0.37
CA 19-9	147.6 ± 468.0	307.0 ± 728.1	.224	−0.26	199.0 ± 562.0	326.8 ± 855.6	.497	−0.18
Span-1	309.5 ± 1711.8	150.2 ± 276.2	.544	0.13	457.3 ± 2107.4	146.6 ± 303.3	.435	0.21
Days from diagnosis to surgery	63.5 (49.3–83.8)	67.0 (56.5–80.5)	.685	0.09	79.0 (50.0–84.3)	67.5 (57.0–77.0)	.447	0.20
Type of hepatectomy (right/left/other)	32/8/4	29/15/1	.131	0.45	25/3/2	20/9/1	.159	0.53
PD (Y/N)	9/35	3/42	.052	0.41	6/24	0/30	.024	0.71
Remnant liver volume (mL)	541.5 ± 118.0	647.4 ± 189.1	.003	−0.67	539.2 ± 104.9	631.2 ± 185.5	.025	−0.61
Vascular resection/reconstruction (Y/N)	10/34	18/27	.078	−0.38	8/22	10/20	.779	−0.15
Estimated blood loss (mL)	1531.6 ± 1857.9	1612.8 ± 1132.3	.804	−0.05	1676.2 ± 2164.2	1671.8 ± 1218.4	.992	0.00
Operative time (minutes)	662.3 ± 149.5	654.7 ± 134.0	.800	0.05	665.3 ± 146.5	645.1 ± 139.8	.586	0.14
Bismuth classification (I/II/III/IV)	0/5/27/12	0/4/29/12	.918	0.09	0/3/21/6	0/4/19/7	.860	0.10
Pathological T stage (1/2/3/4)	1/30/7/6	0/24/5/16	.069	−0.31	0/20/5/5	0/19/2/9	.348	−0.07
Pathological differentiation (well/mod/poor/other)	3/18/21/2	4/17/23/1	.898	0.14	1/12/17/0	3/12/15/0	.774	0.13
Lymph vessel invasion (Y/N/UNK)	20/22/2	19/23/3	.827	0.05	14/16/0	16/14/0	.605	−0.13
Microvascular invasion (Y/N/UNK)	23/19/2	32/10/3	.038	−0.46	20/10/0	22/8/0	.573	−0.15
Perineural invasion (Y/N/UNK)	42/0/2	40/2/3	.093	0.32	30/0/0	30/0/0	NC	0.00
R status (R0/R1, 2)	30/14	21/24	.039	−0.45	21/9	11/19	.009	−0.71
Pathological N stage (0/1)	20/24	20/25	.924	−0.02	14/16	13/17	.795	−0.07
Pathological TNM stage (1/2/3/4)	1/18/18/7	0/13/20/12	.234	−0.30	0/12/14/4	0/10/14/6	.973	−0.14
Postoperative complications (none/Clavien–Dindo classification I/II/III/IV/V)	10/3/6/21/1/3	11/0/5/27/0/2	.250	−0.11	6/1/3/17/1/2	10/0/4/15/0/1	.712	0.27
Length of postoperative hospital stay (d)	37.5 (30.0–67.0)	37.0 (27.5–67.0)	.280	0.23	37.0 (30.8–63.3)	36.0 (26.0–66.8)	.517	0.17
Adjuvant chemotherapy (Y/N/UNK)	15/29/0	18/23/4	.353	−0.47	11/19/0	12/14/4	.472	−0.35
Recurrence (Y/N)	26/18	33/12	.154	−0.30	18/12	21/9	.416	−0.21

Values are presented as mean ± standard deviation or median (IQR).

Alb = albumin, ASA = American Society of Anesthesiologists, BMI = body mass index, CA 19-9 = carbohydrate antigen 19-9, CEA = carcinoembryonic antigen, CRP = C-reactive protein, DUPAN-2 = Duke pancreatic monoclonal antigen type 2, EBD = endoscopic biliary drainage, IQR = interquartile range, N = no, NC = not calculable, NLR = neutrophil-to-lymphocyte ratio, PD = pancreatoduodenectomy, PLR = platelet-to-lymphocyte ratio, PTBD = percutaneous transhepatic biliary drainage, PTPE = percutaneous transhepatic portal embolization, SMD = standardized mean difference, Span-1 = s-pancreas antigen-1, T stage = tumor stage, T-bili = total bilirubin, UNK = unknown, WBC = white blood cell, well = well differentiated, Y = yes.

Before PSM, the sustained CRP group (n = 45) had significantly higher inflammatory values – namely, CRP, NLR, PLR, and CRP/Alb ratio – lower Alb levels, higher T-bili levels prior to surgery, higher carcinoembryonic antigen levels, larger remnant liver volume, and a higher incidence of microvascular invasion or residual tumors compared with the alleviated CRP group (n = 44; Table 4). After PSM, significant differences remained between the 2 groups in CRP prior to surgery, NLR, PLR, Alb, CRP/Alb ratio, T-bili prior to surgery, PD performed at surgery, remnant liver volume, and R status. However, no significant differences were observed between the groups for the other variables, including maximum preoperative CRP, tumor markers, and pathological tumoral factors. The magnitude of the decline from the peak was likewise comparable between the 2 matched groups (sustained, 6.59 ± 4.15 mg/dL; alleviated, 7.77 ± 5.87 mg/dL; *P* = .374), so the groups differed only in the level at which inflammation had settled at surgery (Fig. S1, Supplemental Digital Content 3).

When comparing OS and RFS between the groups before PSM, the sustained CRP group had a significantly shorter OS, but not RFS, compared with the alleviated CRP group (median OS: 29.4 vs 49.8 months, *P* = .038; median RFS: 15.9 vs 26.2 months, *P* = .082; Fig. 1A, B). After PSM, the sustained CRP group had a significantly shorter OS (median OS: 22.6 vs 49.8 months, *P* = .017), whereas the difference in RFS did not reach significance (median RFS: 13.2 vs 25.1 months, *P* = .074; Fig. 1C, D).

**Figure 1.**
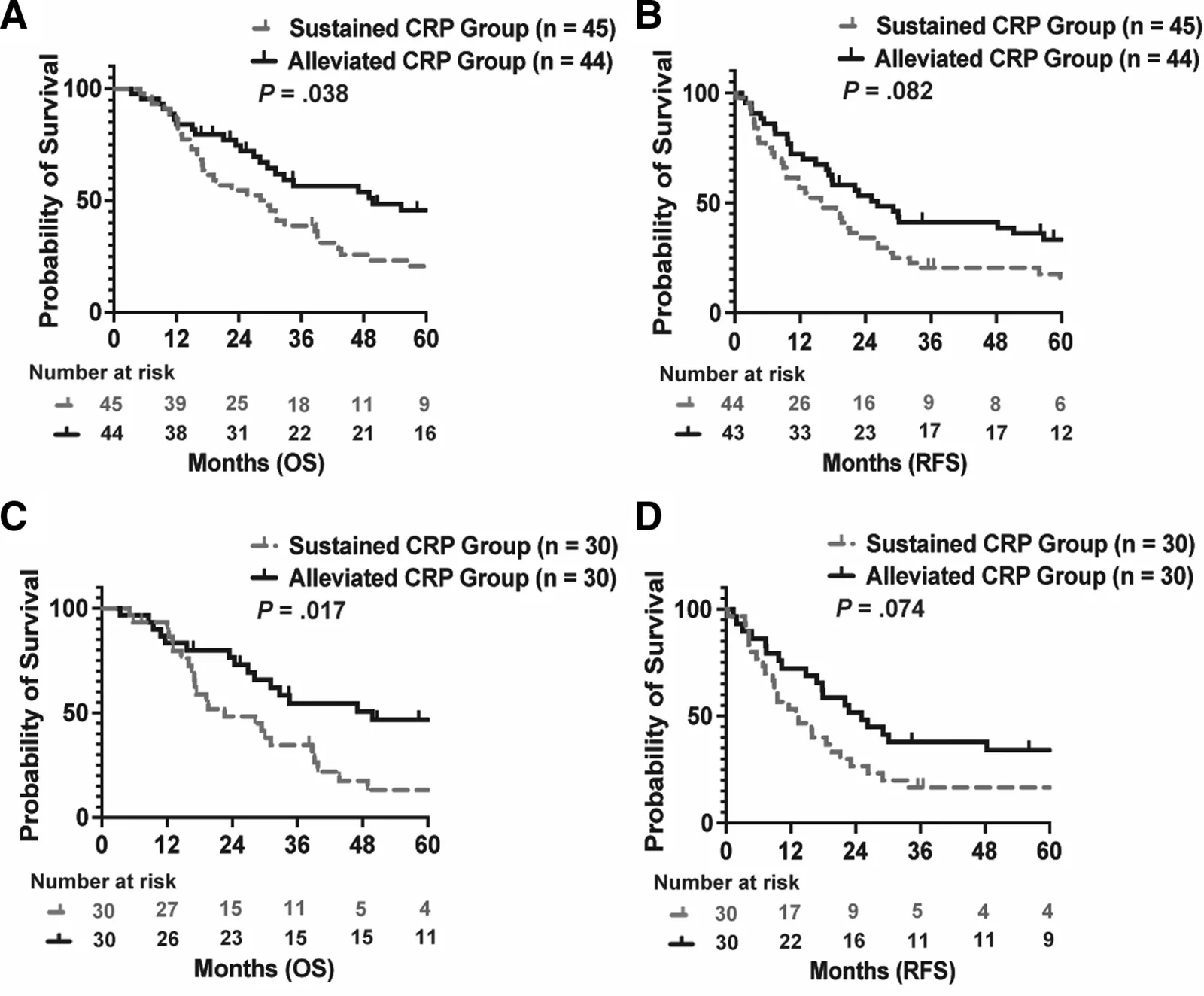
Kaplan–Meier curves for overall survival (A: before, and C: after propensity score matching) and recurrence-free survival (B: before, and D: after propensity score matching) for the CRP groups. CRP = C-reactive protein, OS = overall survival, RFS = recurrence-free survival.

In addition, we conducted a sensitivity multivariate Cox regression analysis for OS to determine whether the CRP group was an independent predictor of survival in P-CCA. In the multivariate analysis, the alleviated CRP group showed better OS (HR, 0.40; 95% CI, 0.21–0.79) than the sustained CRP group, while concomitant PD or R status did not affect OS (Table S4, Supplemental Digital Content 4).

Lastly, because the cause of preoperative inflammation might confound the association between CRP and OS, we repeated the analysis accounting for preoperative cholangitis, which was documented in 90 patients (39 with, 51 without). Adjustment for cholangitis left the estimate for CRP prior to surgery essentially unchanged (from HR, 1.25 to HR, 1.22; 95% CI, 1.05–1.40), and a Cox model stratified on cholangitis yielded an HR of 1.22 (95% CI, 1.05–1.41; *P* = .008). The survival difference between the sustained and alleviated CRP groups was of similar magnitude in patients with and without cholangitis (HR, 1.85 and 1.88, respectively), although neither stratum reached significance; in models stratified on cholangitis, which pool the within-stratum comparisons, the difference was significant both before and after PSM (before: HR, 1.86; *P* = .026; after: HR, 2.09; *P* = .026; Table S5, Supplemental Digital Content 5).

## 4. Discussion

This retrospective cohort study yielded several noteworthy findings. First, consistent with a previous single-institutional retrospective study,^[2]^ we observed a significant association between preoperative cholangitis or systemic inflammation in P-CCA patients and decreased survival. Surprisingly, the maximum preoperative CRP value did not affect survival, suggesting that adequate alleviation of preoperative inflammation may lead to better prognosis after surgery in P-CCA. Second, higher preoperative T-bili was associated with shorter survival and higher recurrence rates, compared with lower preoperative T-bili, even after adequate reduction by preoperative biliary drainage, whereas CRP and other inflammatory markers did not affect the recurrence rate or RFS. Finally, and most importantly, waiting for CRP levels to normalize before surgery was associated with a substantial improvement in patient survival. These findings underscore the critical importance of a reasonable waiting period for inflammation alleviation.

Previous studies have reported several predictors of poor prognosis in resectable P-CCA, such as high preoperative serum T-bili and carbohydrate antigen 19-9 levels, and modified Glasgow prognostic score, consistent with our findings.^[2,18,25]^ Other common inflammatory indexes, including NLR and CRP/Alb ratio, are also predictors of poor prognosis but not of increased recurrence rates. In contrast, the maximum CRP value was not associated with survival in this study. This finding is consistent with a previous study suggesting that the severity of preoperative cholangitis was not associated with poor long-term outcomes in patients with extrahepatic bile duct cancer.^[27]^ Together, these observations may suggest that transient inflammatory episodes occurring during the preoperative course are less prognostically relevant than systemic inflammation that persists immediately before surgery. Interestingly, previous research also supports our findings that preoperative systemic inflammation contributes to worse prognosis without affecting the recurrence rate, possibly due to disturbances in host immunity, which may be critically linked to noncancerous death, including postoperative complications, or decreased survival after recurrence.^[28,29]^ These findings indicate that better outcomes could be achieved by alleviating inflammation before surgery, compared with proceeding to surgery while sustained inflammation is present.

Numerous studies have explored the significance of alleviating jaundice through biliary drainage before resection. Compared with no drainage, endoscopic and/or percutaneous transhepatic biliary drainage showed better short-term surgical outcomes, with improved liver function and reduced risks of postoperative cholangitis and liver failure.^[30]^ However, biliary drainage can lead to procedure-induced adverse events, including hemobilia and cholangitis, as well as potentially increase the rates of postoperative complications, such as infectious diseases.^[20,31,32]^ Additionally, percutaneous biliary drainage is an independent indicator of a poor prognosis following hepatectomies for P-CCA.^[33]^ Overall, there is insufficient evidence to indicate a survival benefit of preoperative biliary drainage for jaundice reduction. Consequently, the National Comprehensive Cancer Network guideline has not established a consensus regarding the optimal approach to biliary drainage and the target value for serum T-bili, making the best timing for resection difficult to determine for practitioners.^[34]^ Our study suggesting the pivotal value of inflammation reduction provides new insight into preoperative care for patients with P-CCA. Alleviating systemic inflammation by treating cholangitis and other inflammation and infections may offer a novel approach in the preoperative management of P-CCA.

The relationship between nononcogenic systemic inflammation and the survival of cancer patients remains unclear. Attie et al demonstrated that even 1 episode of bacterial infection during treatment for colorectal cancer was significantly associated with short OS.^[13]^ Interestingly, patients who experienced 2 or more bacterial infections during treatment had a poorer prognosis than those with 1 or fewer infections. Similar observations have linked postoperative intra-abdominal abscesses to short RFS in patients with resectable hepatocellular carcinomas.^[14]^ Furthermore, several basic studies revealed that systemic inflammation beyond the tumor microenvironment not only facilitates cancer progression but also induces an immunosuppressive environment.^[35-38]^ Our results provide further support for these findings. In this study, we used PSM to standardize tumor characteristics and minimize the possibility that aggressive tumor behavior exacerbating inflammation may be the primary cause of poor survival. Additionally, survival data from a public database analyzed in Gene Expression Profiling Interactive Analysis 2 suggests that messenger RNA expression of CRP in cholangiocarcinomas does not directly impact patient survival (Fig. S2, Supplemental Digital Content 6).^[39]^ These findings further reinforce the crucial role of a reasonable preoperative period for alleviating systemic inflammation.

The limitations of this study include the retrospective, single-institutional study design with a limited number of patients, and no external or higher-level comparator cohort was available. All analyses were performed on complete cases, and no imputation was undertaken. Because the reasons for missingness could not be established retrospectively, we make no assumption that the data were missing at random, and the possibility that the excluded patients differ systematically from those analyzed cannot be dismissed. Another notable limitation was the lack of detailed clinical information on the causes of elevated CRP and their treatment: the severity grade of cholangitis, the results of bile and blood cultures, the class and duration of antimicrobial therapy, and formal sepsis or systemic inflammatory response syndrome scores were not consistently recorded. Given the retrospective design, neither the timing nor the cause of each inflammatory episode was recorded, so we could not match the time course of CRP fluctuations with the treatment administered, nor determine how long before surgery the peak CRP occurred or how completely it had resolved in individual patients. The distinction between a resolved peak and persistent inflammation therefore rests on the 2 measured values alone. Sensitivity analyses were reassuring – adjustment for preoperative cholangitis altered the CRP HR only marginally, and a Cox model stratified on cholangitis gave an essentially unchanged estimate – but the within-stratum analyses were underpowered, and residual confounding by the unmeasured characteristics of the inflammatory episode cannot be excluded.

The study was also underpowered for recurrence. With 52 RFS events in the matched cohort, the smallest HR detectable with 80% power at a 2-sided α of.05 was 2.17, whereas the observed HR was 1.66, and approximately 121 events would have been required; the absence of a significant difference in RFS therefore cannot be read as evidence of no effect. Finally, considering that the time differences from diagnosis to surgery in the alleviated and sustained CRP groups were comparable, questions may arise about the feasibility and potential benefit of waiting longer for CRP reduction to improve prognosis. Furthermore, the target CRP value for surgery remained unclear in this study. A prospective investigation is warranted to determine the impact on survival of preoperative management to achieve specific target CRP levels. Despite these limitations, to our knowledge, this is the first study to highlight the positive impact of preoperative CRP reduction before hepatectomies on the survival of P-CCA patients.

## 5. Conclusions

Our study underscores the association between preoperatively elevated inflammatory markers and short survival in P-CCA patients who undergo resection. Additionally, achieving a marked reduction in preoperative inflammation may improve the prognosis. Although a prospective study is imperative to confirm our findings and identify a practical target CRP value, our results provide valuable insight to enhance preoperative management in P-CCA.

## Acknowledgments

We thank Jane Charbonneau, DVM, from Edanz (https://jp.edanz.com/ac) for editing a draft of this manuscript.

## Author contributions

**Conceptualization:** Yuki Fujii.

**Data curation:** Yuki Fujii, Tatsuhiko Kakisaka, Hirofumi Kamachi.

**Formal analysis:** Yuki Fujii, Ken Imaizumi.

**Investigation:** Yuki Fujii, Hiroki Nakamoto, Tatsuya Orimo, Akihisa Nagatsu, Takeshi Aiyama, Kazuki Wakizaka, Shunsuke Shichi.

**Methodology:** Yuki Fujii, Tatsuhiko Kakisaka, Akinobu Taketomi.

**Supervision:** Tatsuhiko Kakisaka, Hirofumi Kamachi, Akinobu Taketomi.

**Visualization:** Yuki Fujii.

**Writing – original draft:** Yuki Fujii.

**Writing – review & editing:** Tatsuhiko Kakisaka, Hirofumi Kamachi, Hiroki Nakamoto, Tatsuya Orimo, Akihisa Nagatsu, Takeshi Aiyama, Kazuki Wakizaka, Shunsuke Shichi, Ken Imaizumi, Akinobu Taketomi.















## References

[1] Sharma P Yadav S. Demographics, tumor characteristics, treatment, and survival of patients with Klatskin tumors. Ann Gastroenterol. 2018;31:231–6. 10.20524/aog.2018.0233 PMC5825954 29507471

[2] Dumitrascu T Chirita D Ionescu M Popescu I. Resection for hilar cholangiocarcinoma: analysis of prognostic factors and the impact of systemic inflammation on long-term outcome. J Gastrointest Surg. 2013;17:913–24. 10.1007/s11605-013-2144-2 23319395

[3] Lee JW Lee JH Park Y. Prognostic predictability of American Joint Committee on cancer 8th staging system for perihilar cholangiocarcinoma: limited improvement compared with the 7th staging system. Cancer Res Treat. 2020;52:886–95. 10.4143/crt.2020.023 PMC7373861 32164049

[4] Okuno M Ebata T Yokoyama Y. Evaluation of inflammation-based prognostic scores in patients undergoing hepatobiliary resection for perihilar cholangiocarcinoma. J Gastroenterol. 2016;51:153–61. 10.1007/s00535-015-1103-y 26187429

[5] Gaspersz MP Buettner S Roos E. A preoperative prognostic model to predict surgical success in patients with perihilar cholangiocarcinoma. J Surg Oncol. 2018;118:469–76. 10.1002/jso.25174 30132904

[6] Bednarsch J Czigany Z Lurje I. Insufficient future liver remnant and preoperative cholangitis predict perioperative outcome in perihilar cholangiocarcinoma. HPB (Oxford). 2021;23:99–108. 10.1016/j.hpb.2020.04.017 32546423

[7] Dolan RD Lim J McSorley ST Horgan PG McMillan DC. The role of the systemic inflammatory response in predicting outcomes in patients with operable cancer: systematic review and meta-analysis. Sci Rep. 2017;7:16717. 10.1038/s41598-017-16955-5 PMC5711862 29196718

[8] Liu Y Chen S Zheng C. The prognostic value of the preoperative c-reactive protein/albumin ratio in ovarian cancer. BMC Cancer. 2017;17:285. 10.1186/s12885-017-3220-x PMC5399817 28431566

[9] Sanjay P de Figueiredo RS Leaver H. Preoperative serum C-reactive protein levels and post-operative lymph node ratio are important predictors of survival after pancreaticoduodenectomy for pancreatic ductal adenocarcinoma. JOP. 2012;13:199–204. 22406601

[10] Sherry AD Newman NB Anderson JL Osmundson EC. Systemic inflammatory dynamics during chemoradiotherapy predict response, relapse, metastasis, and survival in esophageal carcinoma. J Surg Oncol. 2020;121:303–12. 10.1002/jso.25793 31799692

[11] Ohira M Yoshizumi T Yugawa K. Association of inflammatory biomarkers with long-term outcomes after curative surgery for mass-forming intrahepatic cholangiocarcinoma. Surg Today. 2020;50:379–88. 10.1007/s00595-019-01905-7 31667580

[12] Wu Y Ren F Chai Y. Prognostic value of inflammation-based indexes for intrahepatic cholangiocarcinoma following curative resection. Oncol Lett. 2019;17:165–74. 10.3892/ol.2018.9618 PMC6313216 30655752

[13] Attie R Chinen LT Yoshioka EM Silva MC de Lima VC. Acute bacterial infection negatively impacts cancer specific survival of colorectal cancer patients. World J Gastroenterol. 2014;20:13930–5. 10.3748/wjg.v20.i38.13930 PMC4194575 25320529

[14] Ruan DY Lin ZX Li Y. Poor oncologic outcomes of hepatocellular carcinoma patients with intra-abdominal infection after hepatectomy. World J Gastroenterol. 2015;21:5598–606. 10.3748/wjg.v21.i18.5598 PMC4427684 25987785

[15] Wang Y Fu W Tang Z Meng W Zhou W Li X. Effect of preoperative cholangitis on prognosis of patients with hilar cholangiocarcinoma: a systematic review and meta-analysis. Medicine (Baltimore). 2018;97:e12025. 10.1097/MD.0000000000012025 PMC6112934 30142851

[16] Wiggers JK Groot Koerkamp B Cieslak KP. Postoperative mortality after liver resection for perihilar cholangiocarcinoma: development of a risk score and importance of biliary drainage of the future liver remnant. J Am Coll Surg. 2016;223:321–31.e1. 10.1016/j.jamcollsurg.2016.03.035 PMC4961586 27063572

[17] Nagino M Ebata T Yokoyama Y. Evolution of surgical treatment for perihilar cholangiocarcinoma: a single-center 34-year review of 574 consecutive resections. Ann Surg. 2013;258:129–40. 10.1097/SLA.0b013e3182708b57 23059502

[18] Saito H Noji T Okamura K Tsuchikawa T Shichinohe T Hirano S. A new prognostic scoring system using factors available preoperatively to predict survival after operative resection of perihilar cholangiocarcinoma. Surgery. 2016;159:842–51. 10.1016/j.surg.2015.10.027 26683498

[19] Liu F Li Y Wei Y Li B. Preoperative biliary drainage before resection for hilar cholangiocarcinoma: whether or not? A systematic review. Dig Dis Sci. 2011;56:663–72. 10.1007/s10620-010-1338-7 20635143

[20] Rerknimitr R Angsuwatcharakon P Ratanachu-ek T. Asia-Pacific consensus recommendations for endoscopic and interventional management of hilar cholangiocarcinoma. J Gastroenterol Hepatol. 2013;28:593–607. 10.1111/jgh.12128 23350673

[21] Singer M Deutschman CS Seymour CW. The Third International Consensus Definitions for Sepsis and Septic Shock (Sepsis-3). JAMA. 2016;315:801–10. 10.1001/jama.2016.0287 PMC4968574 26903338

[22] Kim HS Kang MJ Kang J. Practice guidelines for managing extrahepatic biliary tract cancers. Ann Hepatobiliary Pancreat Surg. 2024;28:161–202. 10.14701/ahbps.23-170 PMC11128785 38679456

[23] Aoki Y Noji T Tanaka K. Validation study for prognostic scoring system for perihilar cholangiocarcinoma surgery using preoperative factors. Langenbecks Arch Surg. 2023;408:430. 10.1007/s00423-023-03145-3 37938415

[24] Hu HJ Jin YW Zhou RX. Clinical value of inflammation-based prognostic scores to predict the resectability of hyperbilirubinemia patients with potentially resectable hilar cholangiocarcinoma. J Gastrointest Surg. 2019;23:510–7. 10.1007/s11605-018-3892-9 30076591

[25] Lee SH Choi GH Han DH Kim KS Choi JS Rho SY. Chronological analysis of surgical and oncological outcomes after the treatment of perihilar cholangiocarcinoma. Ann Hepatobiliary Pancreat Surg. 2021;25:62–70. 10.14701/ahbps.2021.25.1.62 PMC7952679 33649256

[26] Oshiro Y Sasaki R Fukunaga K. Inflammation-based prognostic score is a useful predictor of postoperative outcome in patients with extrahepatic cholangiocarcinoma. J Hepatobiliary Pancreat Sci. 2013;20:389–95. 10.1007/s00534-012-0550-6 23138964

[27] Akita M Ajiki T Matsumoto T. Preoperative cholangitis affects survival outcome in patients with extrahepatic bile duct cancer. J Gastrointest Surg. 2017;21:983–9. 10.1007/s11605-017-3388-z 28290140

[28] Yoshikawa N Yoshida K Tamauchi S. The preoperative prognostic nutritional index for the prediction of outcomes in patients with early-stage ovarian clear cell carcinoma. Sci Rep. 2020;10:7135. 10.1038/s41598-020-64171-5 PMC7189228 32346076

[29] Seo YJ Kim KE Jeong WK Baek SK Bae SU. Effect of preoperative pan-immune-inflammation value on clinical and oncologic outcomes after colorectal cancer surgery: a retrospective study. Ann Surg Treat Res. 2024;106:169–77. 10.4174/astr.2024.106.3.169 PMC10902619 38435496

[30] Sewnath ME Karsten TM Prins MH Rauws EJ Obertop H Gouma DJ. A meta-analysis on the efficacy of preoperative biliary drainage for tumors causing obstructive jaundice. Ann Surg. 2002;236:17–27. 10.1097/00000658-200207000-00005 PMC1422544 12131081

[31] Ferrero A Lo Tesoriere R Vigano L Caggiano L Sgotto E Capussotti L. Preoperative biliary drainage increases infectious complications after hepatectomy for proximal bile duct tumor obstruction. World J Surg. 2009;33:318–25. 10.1007/s00268-008-9830-3 19020929

[32] Maguchi H Takahashi K Katanuma A. Preoperative biliary drainage for hilar cholangiocarcinoma. J Hepatobiliary Pancreat Surg. 2007;14:441–6. 10.1007/s00534-006-1192-3 17909711

[33] Young AL Igami T Senda Y. Evolution of the surgical management of perihilar cholangiocarcinoma in a Western centre demonstrates improved survival with endoscopic biliary drainage and reduced use of blood transfusion. HPB (Oxford). 2011;13:483–93. 10.1111/j.1477-2574.2011.00328.x PMC3133715 21689232

[34] National Comprehensive Cancer Network. NCCN clinical practice guidelines in oncology: biliary tract cancers. Version 3.2023. https://www.nccn.org/professionals/physician_gls/pdf/btc.pdf. Accessed December 24, 2023. 10.6004/jnccn.2025.0042 40930144

[35] Liu X Yin L Shen S Hou Y. Inflammation and cancer: paradoxical roles in tumorigenesis and implications in immunotherapies. Genes Dis. 2023;10:151–64. 10.1016/j.gendis.2021.09.006 PMC10066281 37013041

[36] Llitjos JF Auffray C Alby-Laurent F. Sepsis-induced expansion of granulocytic myeloid-derived suppressor cells promotes tumour growth through Toll-like receptor 4. J Pathol. 2016;239:473–83. 10.1002/path.4744 27178223

[37] Ruan WS Feng MX Xu J. Early activation of myeloid-derived suppressor cells participate in sepsis-induced immune suppression via PD-L1/PD-1 axis. Front Immunol. 2020;11:1299. 10.3389/fimmu.2020.01299 PMC7347749 32719675

[38] Kohlhapp FJ Huelsmann EJ Lacek AT. Non-oncogenic acute viral infections disrupt anti-cancer responses and lead to accelerated cancer-specific host death. Cell Rep. 2016;17:957–65. 10.1016/j.celrep.2016.09.068 PMC5589518 27760326

[39] Tang Z Kang B Li C. GEPIA2: an enhanced web server for large-scale expression profiling and interactive analysis. Nucleic Acids Res. 2019;47:W556–60. 10.1093/nar/gkz430 PMC6602440 31114875

