# Supplementary material for: Preoperative sustained C-reactive protein elevation shortens patient survival after liver resection for perihilar cholangiocarcinoma: A retrospective cohort study with propensity score analyses
Source: Medicine (Baltimore). 2026 Oct 30;105(44):e51245. doi: 10.1097/MD.0000000000051245 (PMC13727158; doi:10.1097/MD.0000000000051245)
Supplement: Supplementary file 1 [file medi-105-e51245-s001.docx]

**Supplemental Table 1. Alternative parameterizations of CRP prior to surgery associated with overall survival (n = 100).**

| **Parameterization** | **Univariate HR (95% CI)** | ***P-value*** | **Multivariate HR**  **(95% CI)** | ***P-value*** |
| --- | --- | --- | --- | --- |
| Per 1 mg/dL | 1.22 (1.07–1.39) | .002 | 1.18 (1.01–1.38) | .039 |
| Per 1 SD (1.85 mg/dL) | 1.45 (1.14–1.84) | .002 | 1.36 (1.02–1.82) | .039 |
| Per doubling (log₂) | 1.14 (1.03–1.27) | .015 | 1.13 (1.00–1.27) | .052 |
| Per 1 unit of ln(CRP) | 1.21 (1.04–1.42) | .015 | 1.19 (1.00–1.41) | .052 |
| Tertile 1 (≤ 0.20 mg/dL) | Reference |  | Reference |  |
| Tertile 2 (0.21–0.94 mg/dL) | 1.27 (0.70–2.28) | .430 | 1.39 (0.70–2.76) | .350 |
| Tertile 3 (> 0.94 mg/dL) | 1.93 (1.09–3.42) | .025 | 1.88 (1.01–3.50) | .048 |
| Trend across tertiles | 1.40 (1.05–1.87) | .023 | 1.37 (1.01–1.86) | .045 |

Adjusted for age, sex, vascular resection, Bismuth classification, R status and pathological TNM stage. Abbreviations: CI, confidence interval; CRP, C-reactive protein; HR, hazard ratio; SD, standard deviation.
