# Supplementary material for: Preoperative sustained C-reactive protein elevation shortens patient survival after liver resection for perihilar cholangiocarcinoma: A retrospective cohort study with propensity score analyses
Source: Medicine (Baltimore). 2026 Oct 30;105(44):e51245. doi: 10.1097/MD.0000000000051245 (PMC13727158; doi:10.1097/MD.0000000000051245)
Supplement: Supplementary file 2 [file medi-105-e51245-s002.docx]

| **Supplemental Table 3.** Locations of Recurrence | |
| --- | --- |
| **Location** | **Patients, Number (%)** |
| Locoregional | 21 (21%) |
| Peritoneum | 15 (15%) |
| Liver | 12 (12%) |
| Extra-regional lymph node | 6 (6%) |
| Lung | 2 (2%) |
| Bone | 2 (2%) |
| Multiple | 8 (8%) |
| Others | 2 (2%) |
