# Supplementary material for: Preoperative sustained C-reactive protein elevation shortens patient survival after liver resection for perihilar cholangiocarcinoma: A retrospective cohort study with propensity score analyses
Source: Medicine (Baltimore). 2026 Oct 30;105(44):e51245. doi: 10.1097/MD.0000000000051245 (PMC13727158; doi:10.1097/MD.0000000000051245)
Supplement: Supplementary file 3 [file medi-105-e51245-s004.docx]

| **Supplemental Table 4.** Univariate and Multivariate Cox Regression Analyses of Overall Survival after Propensity Score Matching (n = 60) | | | | | | | | | | |
| --- | --- | --- | --- | --- | --- | --- | --- | --- | --- | --- |
|  |  | **Univariate** | | | |  | **Multivariate** | | | |
| **Variable** |  | **Hazard ratio** | **95% CI** | | ***P*-value** |  | **Hazard ratio** | **95% CI** | | ***P*-value** |
| CRP Group | Sustained CRP group | Reference | | | .020 |  | Reference | | | .006 |
|  | Alleviated CRP group | 0.49 | 0.27 | 0.89 |  |  | 0.40 | 0.21 | 0.79 |  |
| PD | No | Reference | | | .283 |  | Reference | | | .062 |
|  | Yes | 1.67 | 0.65 | 4.26 |  |  | 2.75 | 0.95 | 7.96 |  |
| R status | R0 | Reference | | | .981 |  | Reference | | | .962 |
|  | R1/R2 | 1.01 | 0.56 | 1.81 |  |  | 1.01 | 0.55 | 1.88 |  |

CI, confidence interval; CRP, C-reactive protein; PD, pancreatoduodenectomy; R status, remnant tumor status
