# Supplementary material for: Preoperative sustained C-reactive protein elevation shortens patient survival after liver resection for perihilar cholangiocarcinoma: A retrospective cohort study with propensity score analyses
Source: Medicine (Baltimore). 2026 Oct 30;105(44):e51245. doi: 10.1097/MD.0000000000051245 (PMC13727158; doi:10.1097/MD.0000000000051245)
Supplement: Supplementary file 4 [file medi-105-e51245-s005.docx]

**Supplemental Table 5. Sensitivity Analyses Based on the Presence of Preoperative Cholangitis**

| **Analysis** | **n** | **Events** | **HR (95% CI)** | ***P-value*** |
| --- | --- | --- | --- | --- |
| ***Effect of adjustment for cholangitis on CRP prior to surgery (per 1 mg/dL)*** |  |  |  |  |
| CRP alone (patients with cholangitis data) | 90 | 68 | 1.25 (1.09–1.44) | .002 |
| CRP + preoperative cholangitis | 90 | 68 | 1.22 (1.05–1.40) | .008 |
| Multivariate modelᵃ | 100 | 77 | 1.18 (1.01–1.38) | .039 |
| Multivariate modelᵃ + preoperative cholangitis | 90 | 68 | 1.10 (0.92–1.31) | .290 |
| ***CRP prior to surgery (per 1 mg/dL) within strata of cholangitis*** |  |  |  |  |
| Cholangitis present | 39 | 34 | 1.24 (1.05–1.47) | .014 |
| Cholangitis absent | 51 | 34 | 1.17 (0.88–1.53) | .280 |
| Cox model stratified on cholangitis (pooled) | 90 | 68 | 1.22 (1.05–1.41) | .008 |
| ***Sustained versus alleviated CRP group within strata of cholangitis*** |  |  |  |  |
| Cholangitis present (median OS 19.6 vs 47.0 months) | 37 | 33 | 1.85 (0.90–3.81) | .091 |
| Cholangitis absent (median OS 31.1 vs 73.0 months) | 42 | 26 | 1.88 (0.81–4.33) | .136 |
| Stratified on cholangitis, pooled (median OS 28.2 vs 55.2 months) | 79 | 59 | 1.86 (1.08–3.22) | .026 |
| Matched cohort, cholangitis present (median OS 19.2 vs 49.8 months) | 25 | 21 | 2.75 (1.06–7.15) | .031 |
| Matched cohort, stratified on cholangitis (median OS 19.6 vs 49.8 months) | 55 | 43 | 2.09 (1.09–3.98) | .026 |

ᵃ Adjusted for age, sex, vascular resection, Bismuth classification, R status and pathological TNM stage. Abbreviations: CI, confidence interval; CRP, C-reactive protein; HR, hazard ratio; OS, overall survival.
