# Supplementary material for: Preoperative sustained C-reactive protein elevation shortens patient survival after liver resection for perihilar cholangiocarcinoma: A retrospective cohort study with propensity score analyses
Source: Medicine (Baltimore). 2026 Oct 30;105(44):e51245. doi: 10.1097/MD.0000000000051245 (PMC13727158; doi:10.1097/MD.0000000000051245)
Supplement: Supplementary file 5 [file medi-105-e51245-s007.docx]

**Supplemental Table 2. Alternative Parameterizations of Maximum Preoperative CRP Associated with Overall Survival (n = 100).**

| **Parameterization** | **Univariate HR (95% CI)** | ***P-value*** | **Multivariate HR**  **(95% CI)** | ***P-value*** |
| --- | --- | --- | --- | --- |
| Per 1 mg/dL | 1.03 (0.99–1.07) | .203 | 1.02 (0.98–1.06) | .383 |
| Per 1 SD (6.03 mg/dL) | 1.17 (0.92–1.48) | .203 | 1.12 (0.86–1.46) | .383 |
| Per doubling (log₂) | 1.08 (0.95–1.22) | .255 | 1.04 (0.91–1.18) | .567 |
| Per 1 unit of ln(CRP) | 1.11 (0.93–1.33) | .255 | 1.06 (0.88–1.27) | .567 |
| Tertile 1 (≤ 4.82 mg/dL) | Reference |  |  |  |
| Tertile 2 (4.83–9.78 mg/dL) | 0.98 (0.56–1.71) | .937 | 0.83 (0.44–1.57) | .570 |
| Tertile 3 (> 9.78 mg/dL) | 1.29 (0.75–2.23) | .363 | 1.25 (0.70–2.24) | .448 |
| Trend across tertiles | 1.14 (0.86–1.50) | .369 | 1.12 (0.83–1.51) | .463 |

Adjusted for age, sex, vascular resection, Bismuth classification, R status and pathological TNM stage. Abbreviations: CI, confidence interval; CRP, C-reactive protein; HR, hazard ratio; SD, standard deviation.
